# Supplementary material for: Genetically Encoded Biosensors to Monitor Intracellular Reactive Oxygen and Nitrogen Species and Glutathione Redox Potential in Skeletal Muscle Cells
Source: Int J Mol Sci. 2021 Oct 8;22(19):10876. doi: 10.3390/ijms221910876 (PMC8509583; doi:10.3390/ijms221910876)

Supplementary - Figure 1(a)

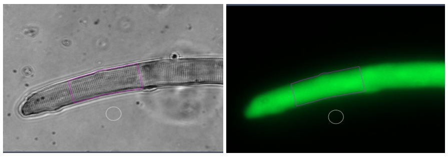

Fluorescence Emis.520 (Exc.488)

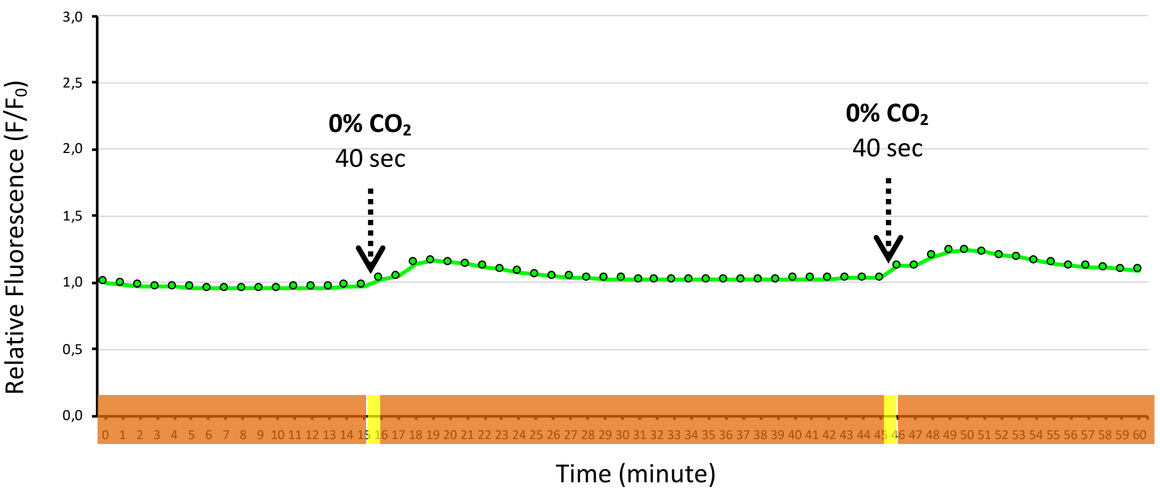

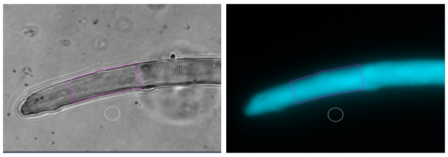

Fluorescence Emis.520 (Exc.420)

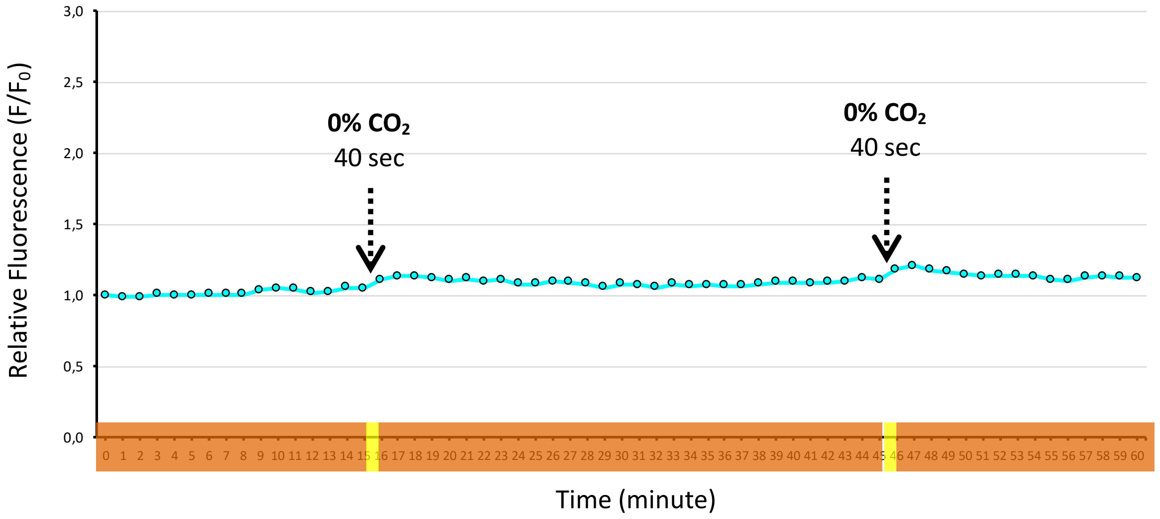

Fluorescence Emis.520 (Exc.488) / Fluorescence Emis.520 (Exc.420)

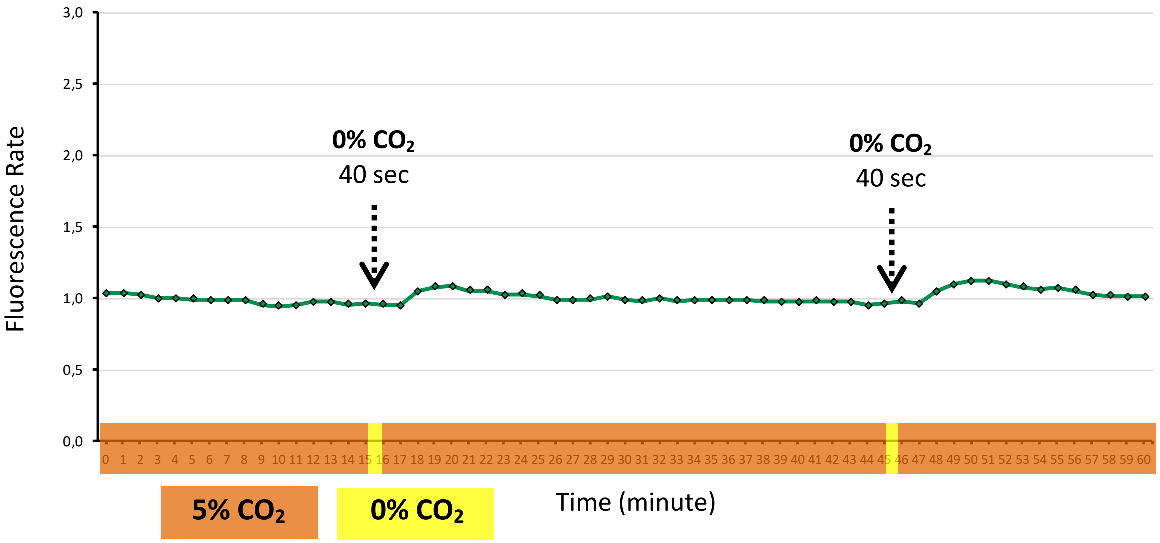

Supplementary - Figure 1(b)

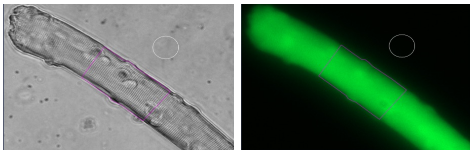

Fluorescence Emis.520 (Exc.488)

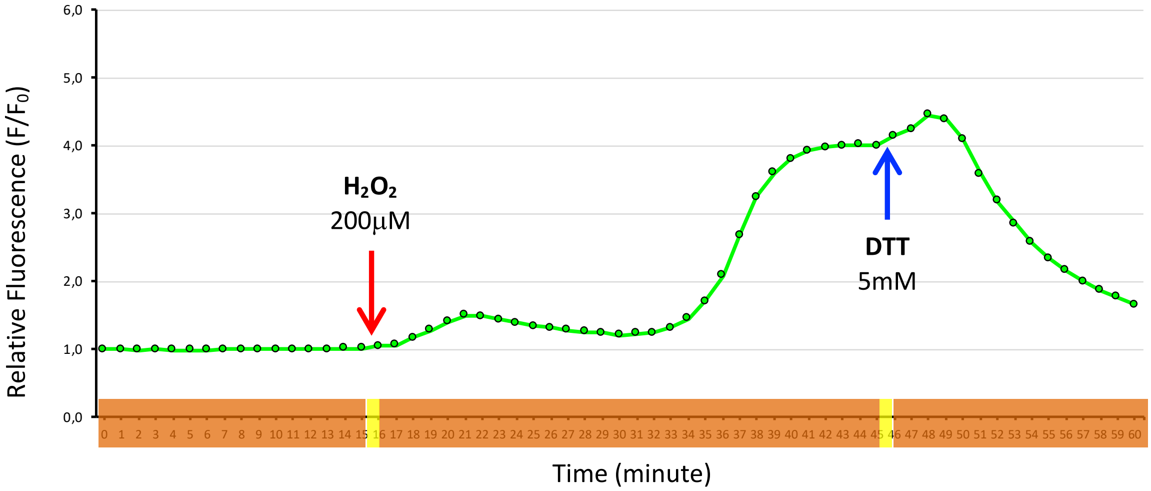

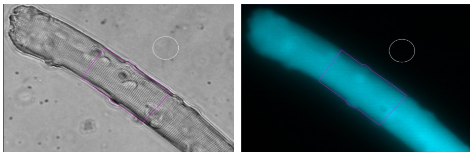

Fluorescence Emis.520 (Exc.420)

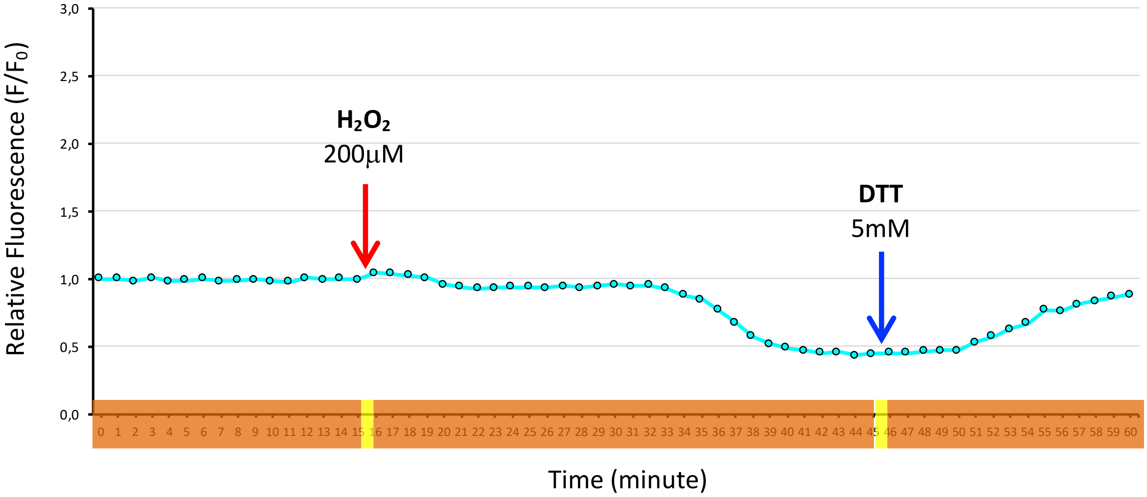

Fluorescence Emis.520 (Exc.488) / Fluorescence Emis.520 (Exc.420)

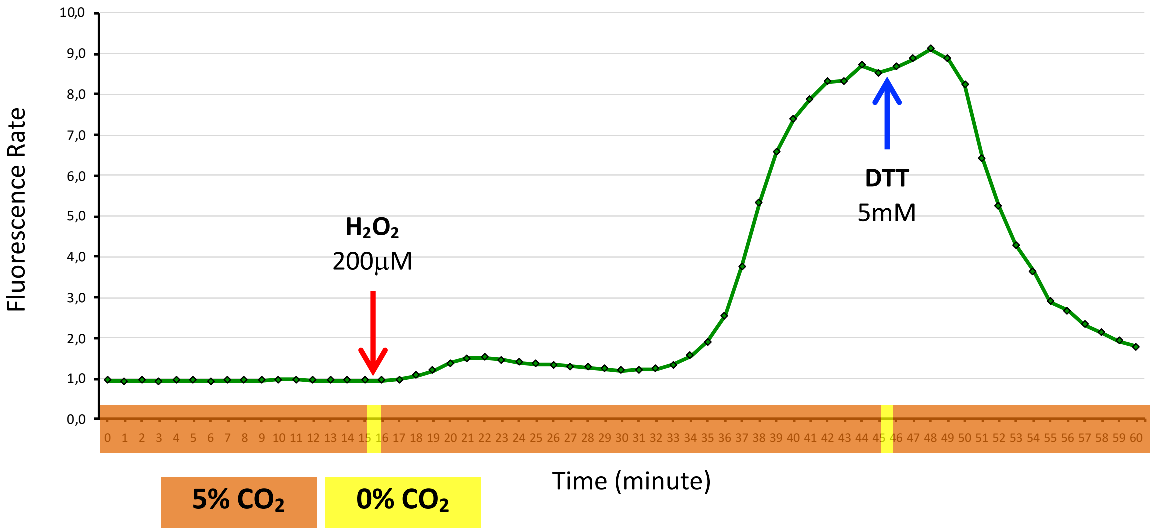

Supplementary - Figure 2

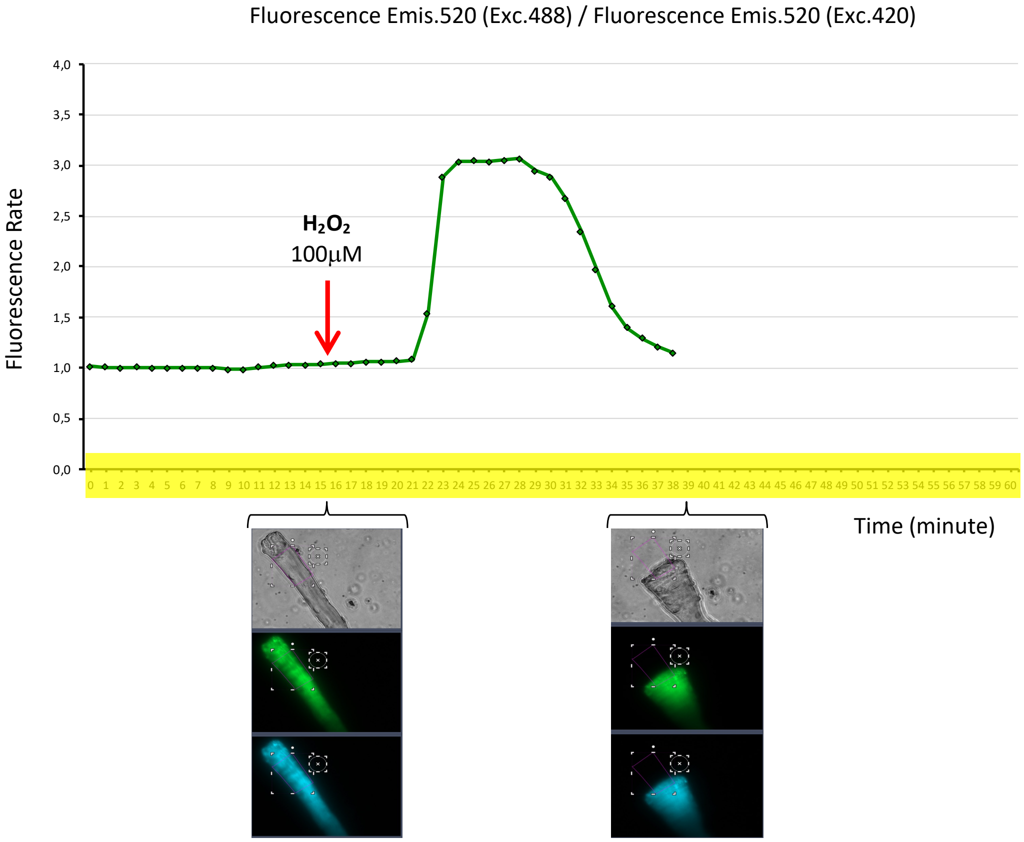

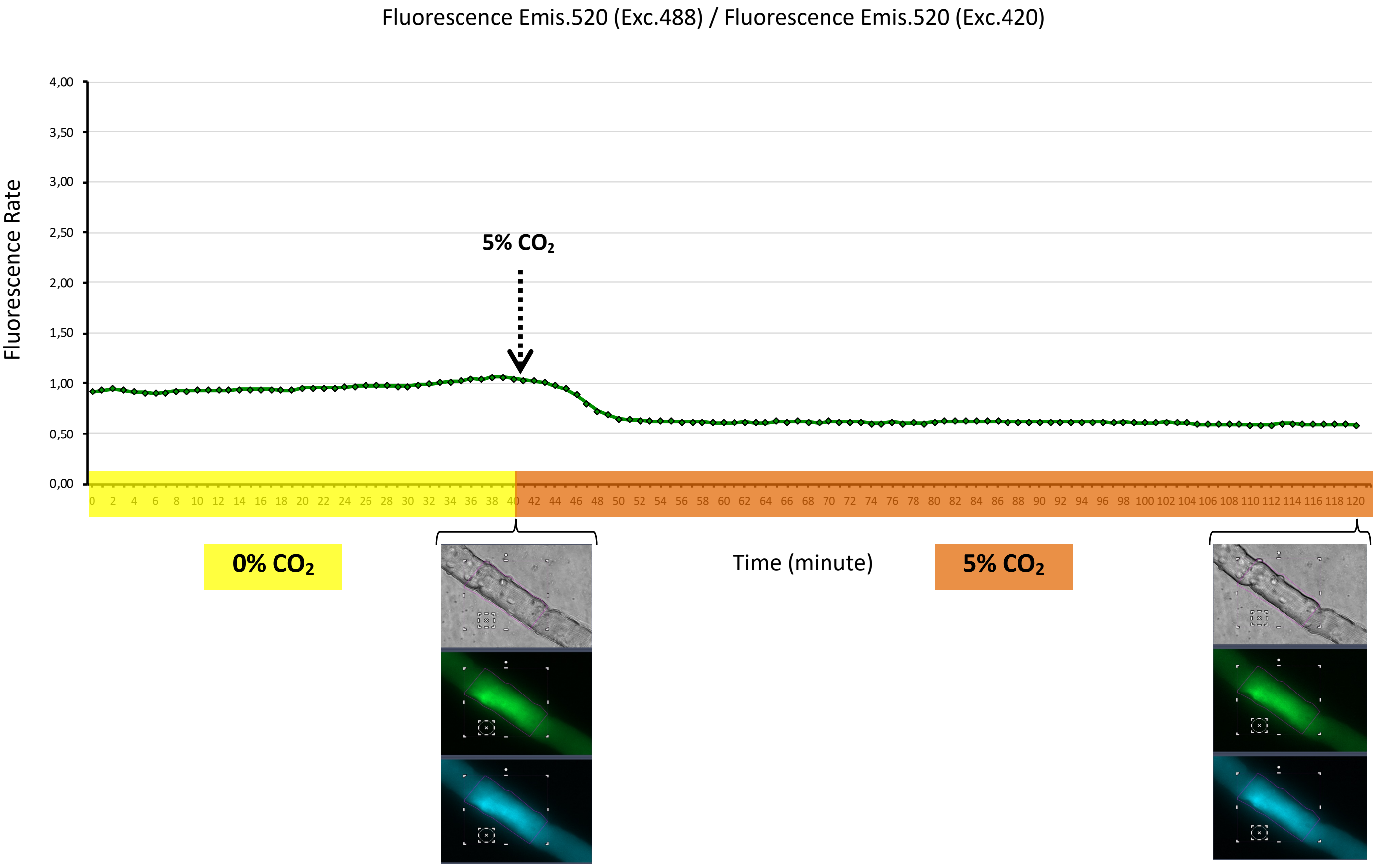

Supplementary - Figure 3

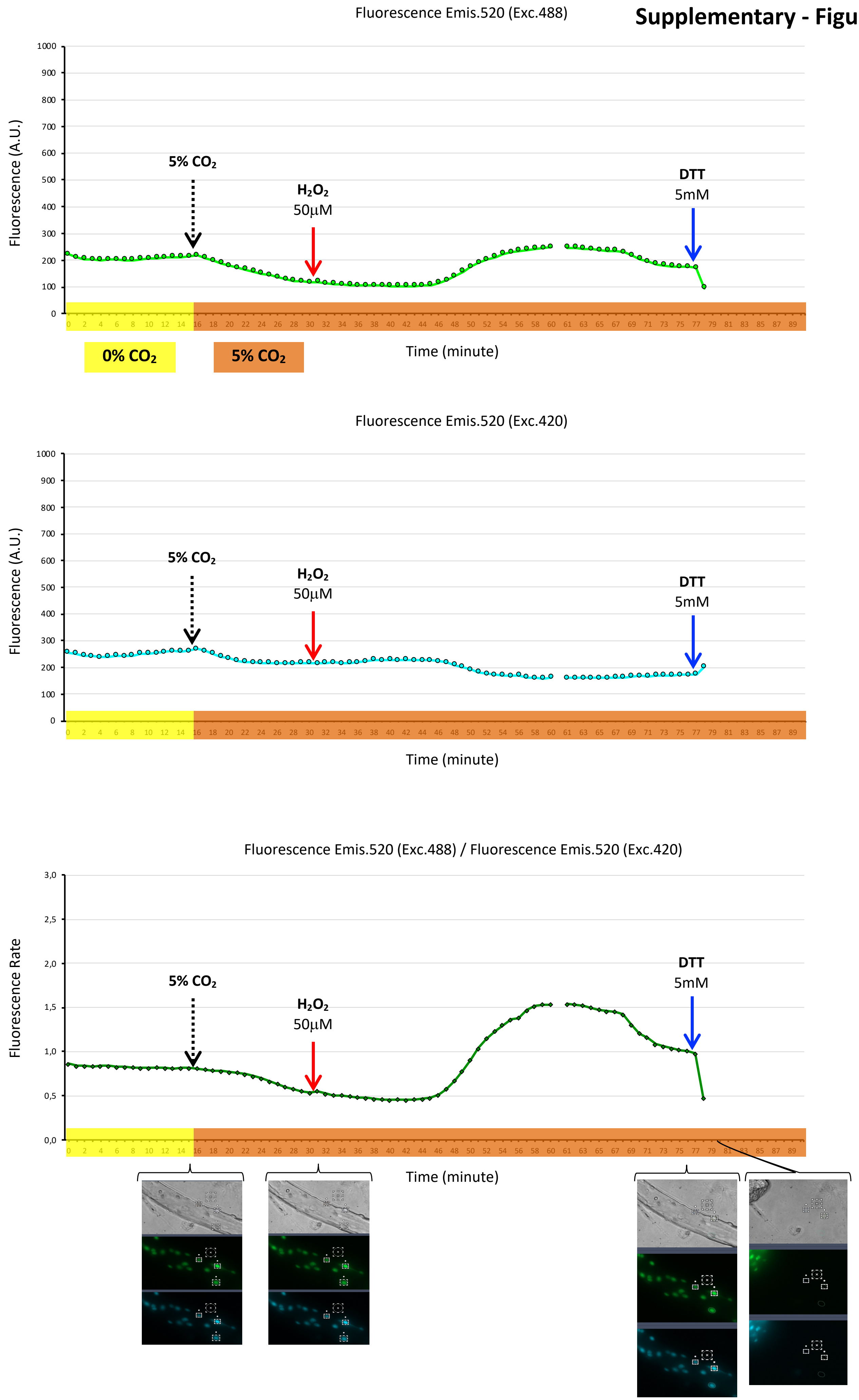

Supplementary - Figure 4(a)

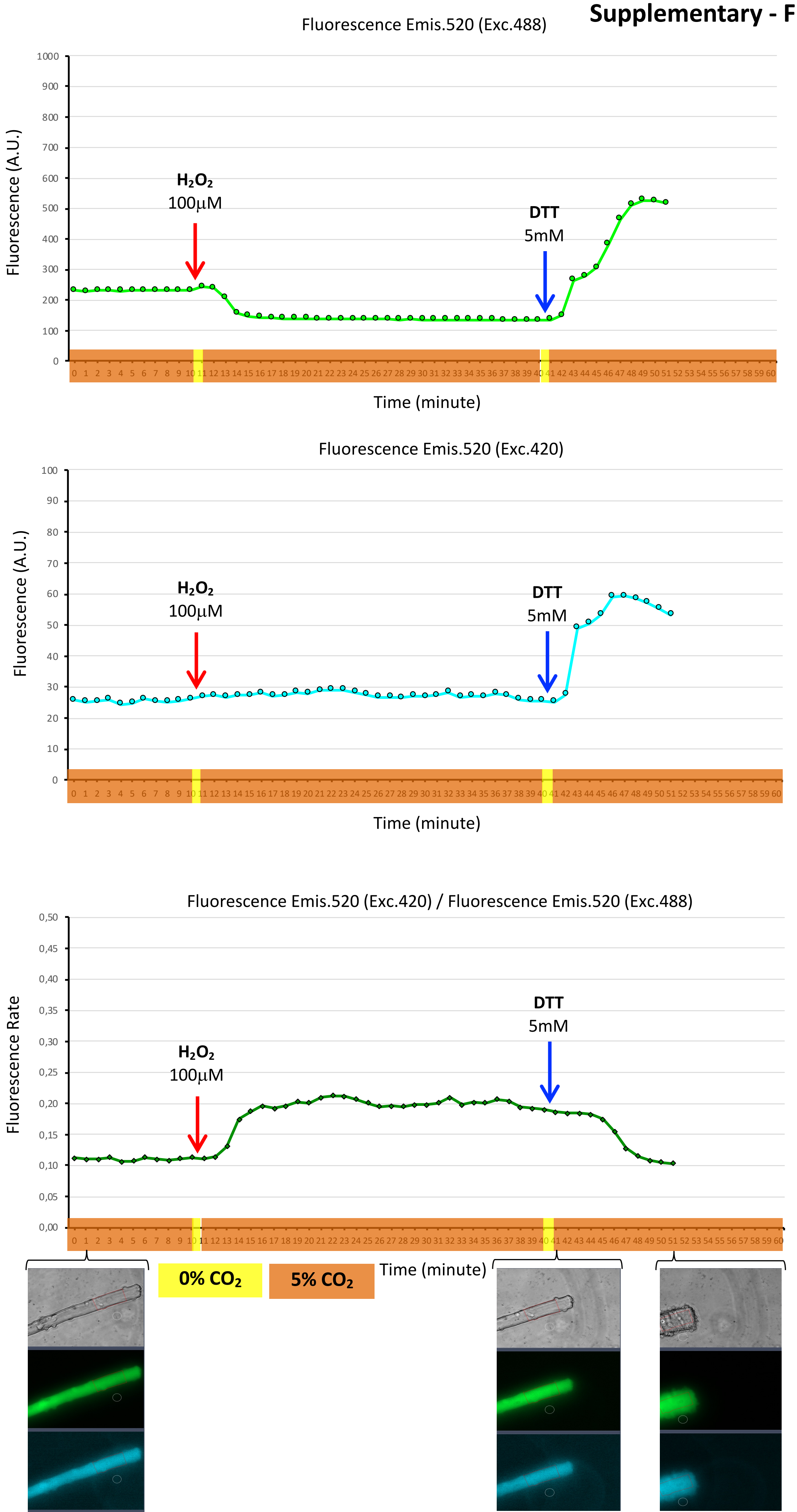

Supplementary - Figure 4(b)

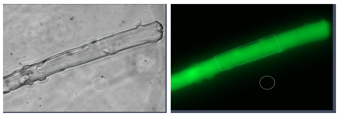

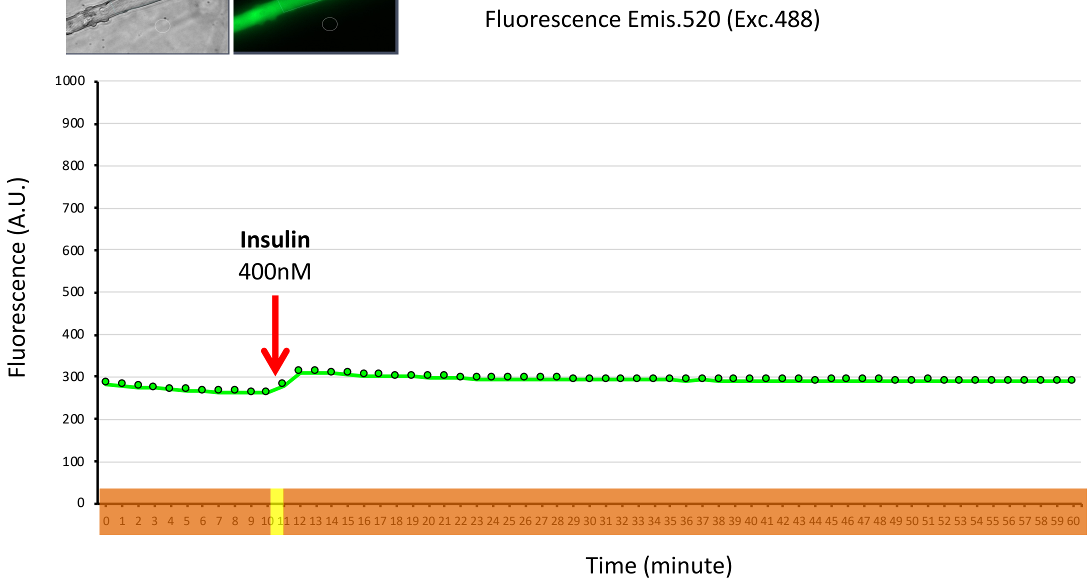

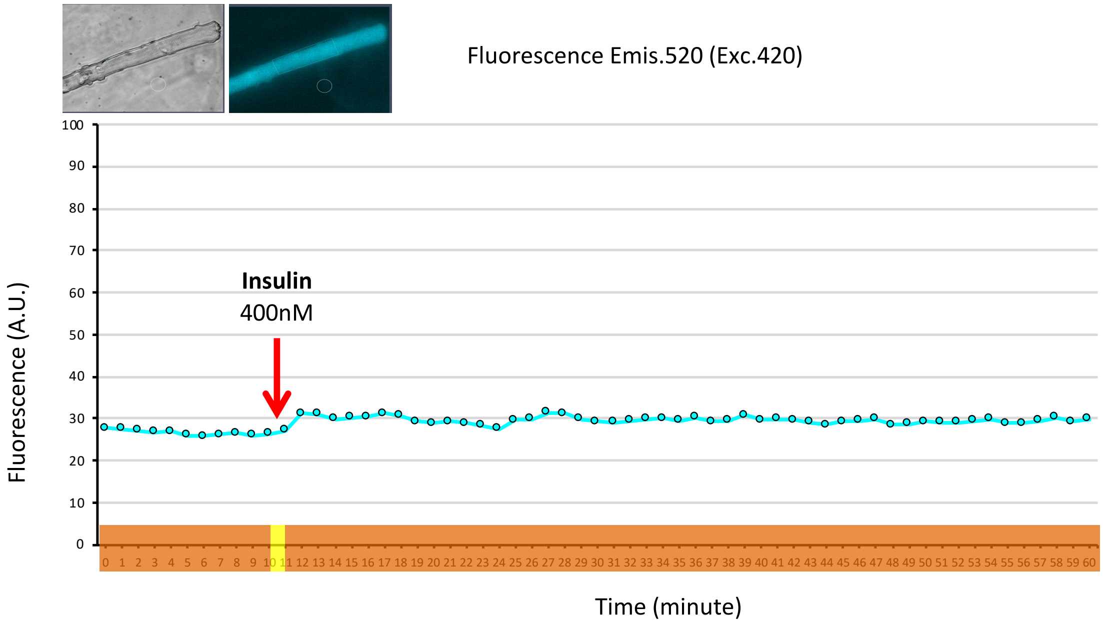

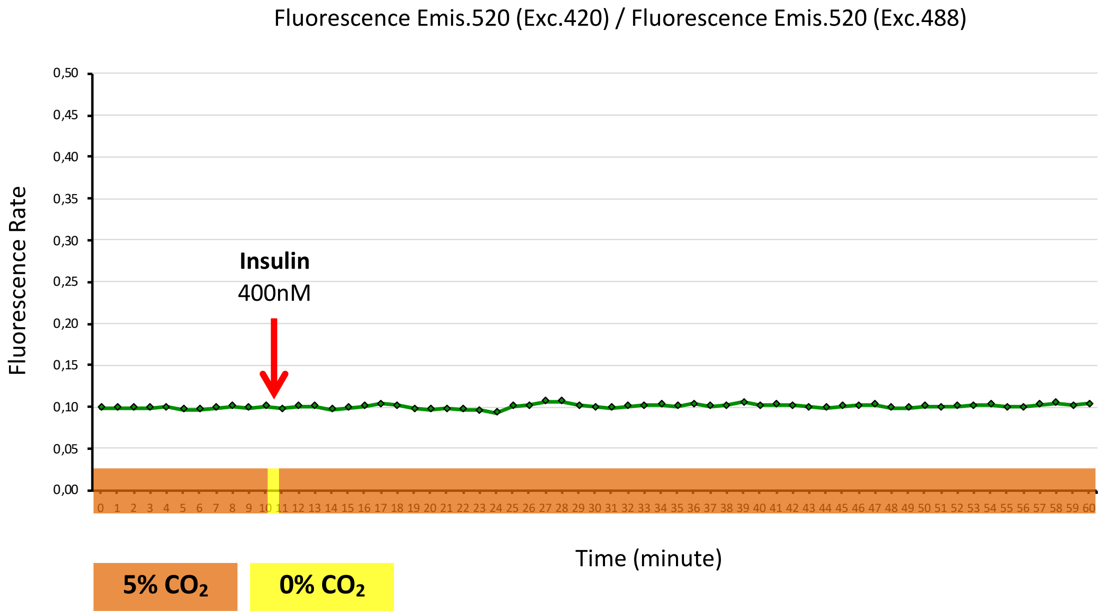

Supplementary - Figure 5

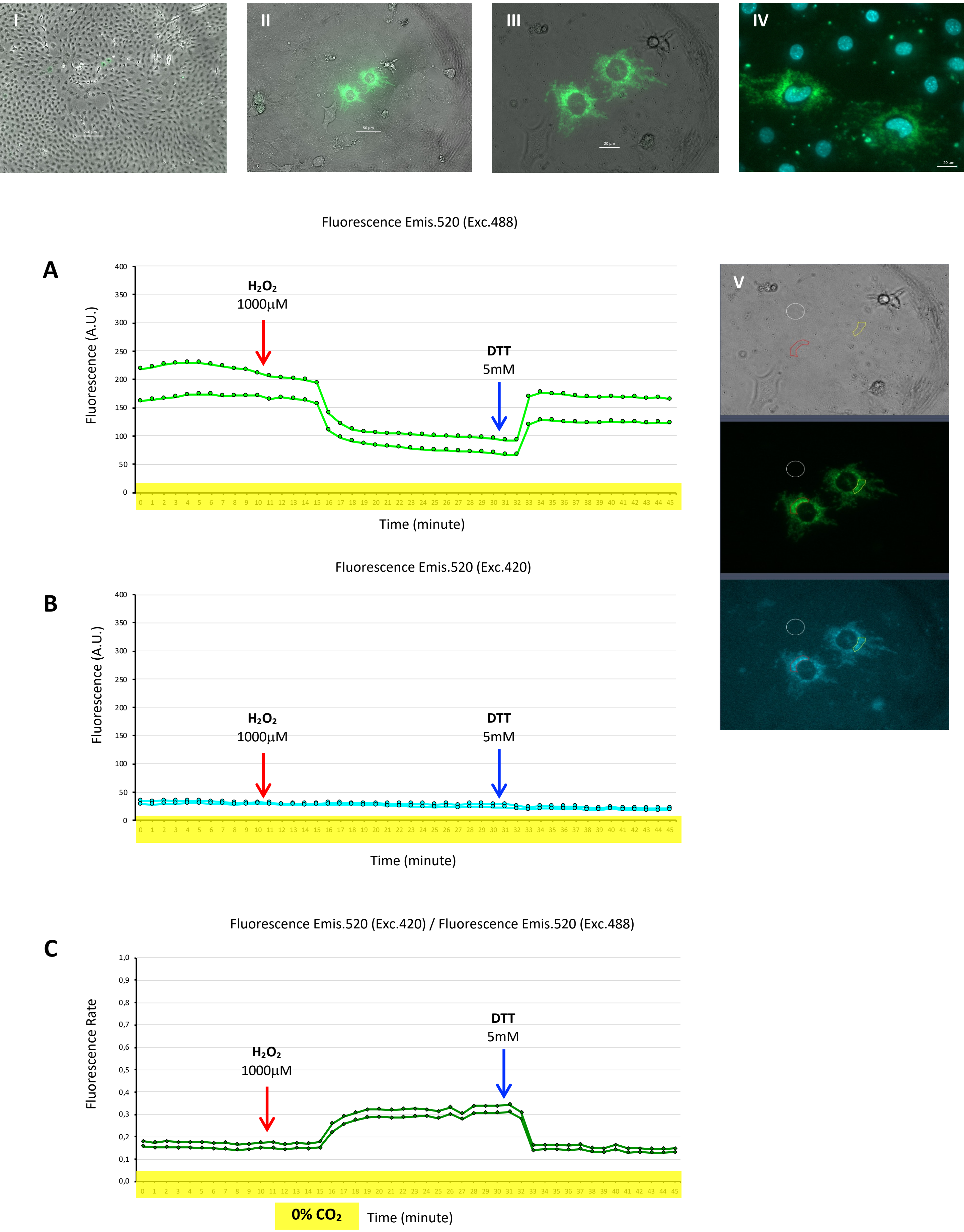

Supplementary - Figure 6

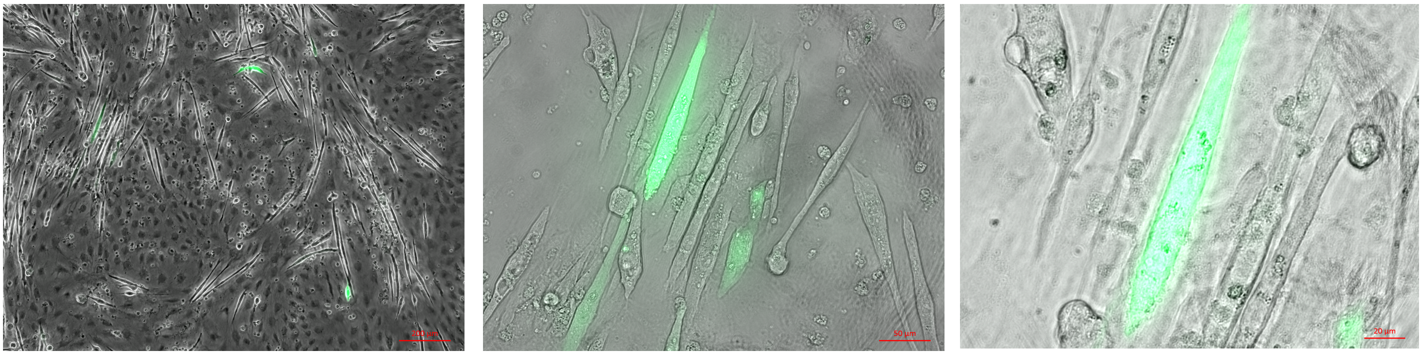

5× **20×** **40×**

Supplementary - Figure 7

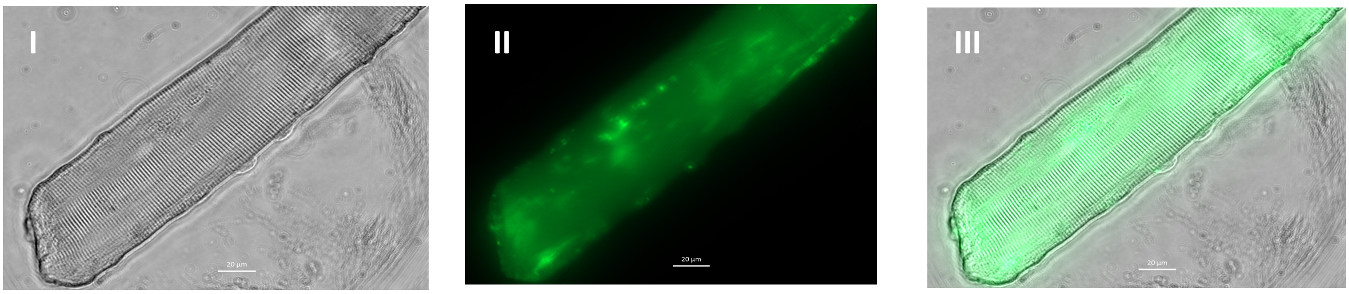

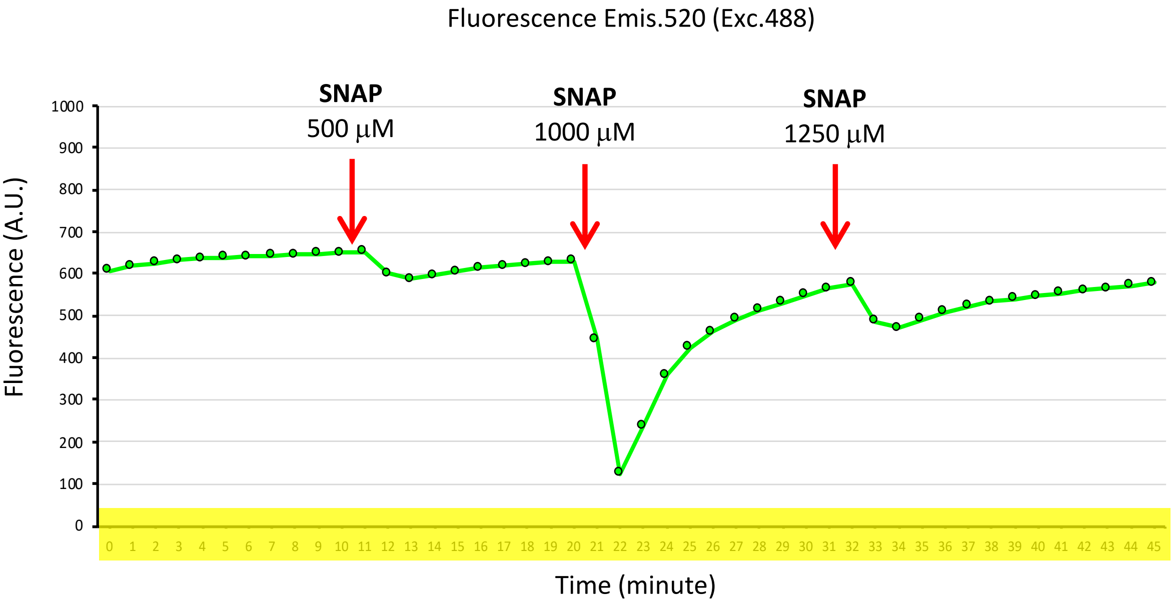

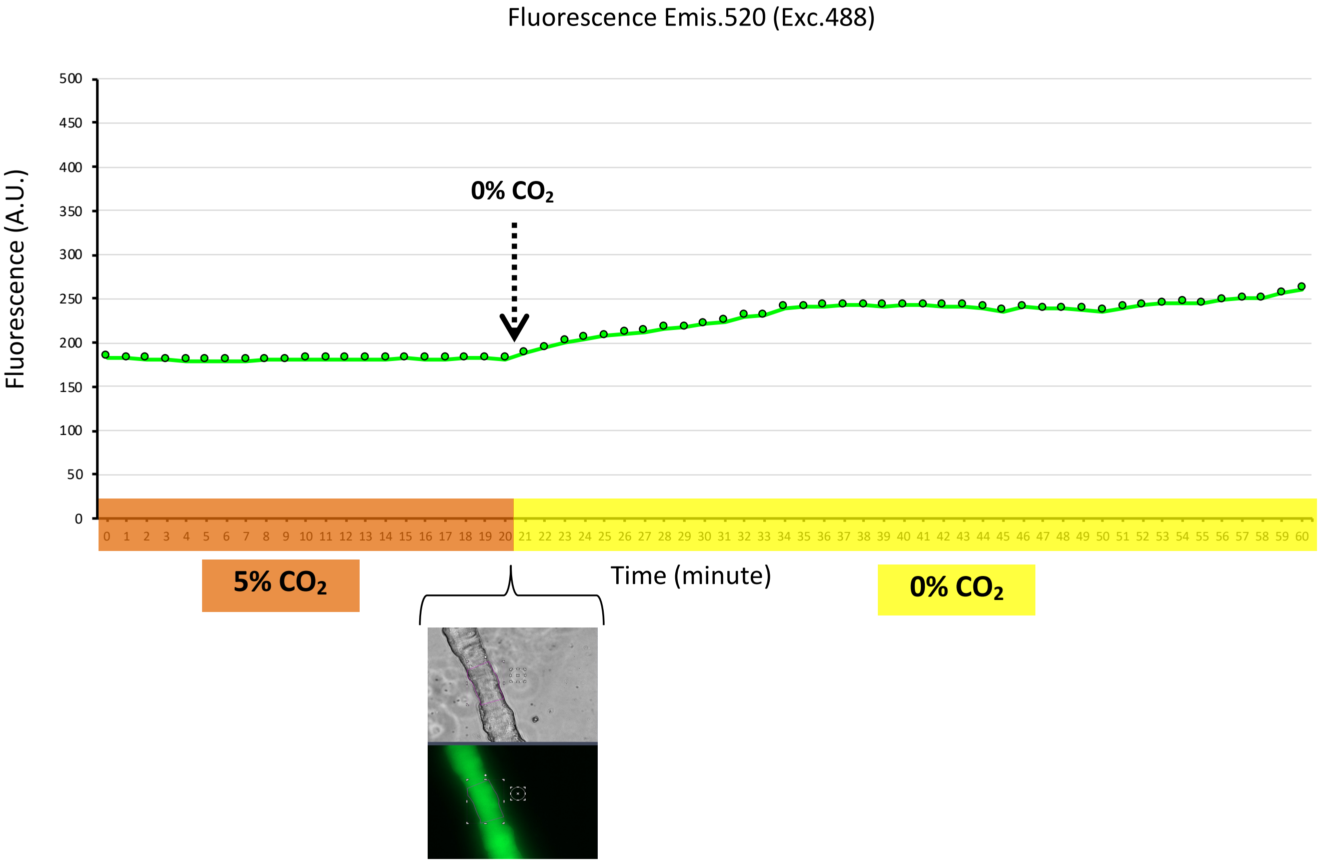

pH assessment of cell culture medium exposed at different CO_2 environmental concentration. Correlation between fluctuations of CO_2 environmental concentration and changes of pH of cell culture medium.

We constantly observed that sensitivity of biosensors to pH was present when experiments were undertaken. We realised the pH effect on fluorescence emitted by biosensors when we disrupted the CO_2 atmosphere from 0 to 5% CO_2 and from 5 to 0% CO_2. When this CO_2 disruption occurred, it was rapidly reflected on the fluorescence emitted by biosensors, such as are shown in figures 1(a), 2(b), 3 and 4. A rapid disruption (40 seconds) of CO_2 atmosphere produced changes in biosensor fluorescence. In addition, to assess that CO_2 affects pH we undertook a test experiment based in a colorimetric pH indicator, Phenol Red, which is used broadly in culture medium as pH indicator. Thus, image below shows three plates with cell culture medium that were placed at different CO_2 atmosphere and temperature. Central plate was at 5% CO_2 atmosphere, and medium shows an orange - soft pink colour, which indicates a neutral pH (7,0-7,5). However, plates, right and left hand-side, were at CO_2 atmosphere around 0% CO_2 (room CO_2 atmosphere), and the colour of the medium was strong pink or violet, which indicated a basic pH (pH>8,0). These situations concerning pH can be extrapolated for the time course experiments that are presented on figures. Thus, when the disruption of CO_2 atmosphere occurred for 40 seconds or longer time, the fluorescence of biosensors changed. As it is stated in the discussion and supported by references, the fluorochromes of biosensors, YFP and GFP, are sensitive to pH. This drives to conclude, that the changes in biosensor fluorescence when CO_2 atmosphere was modified were evoked by pH fluctuations of cell culture medium.

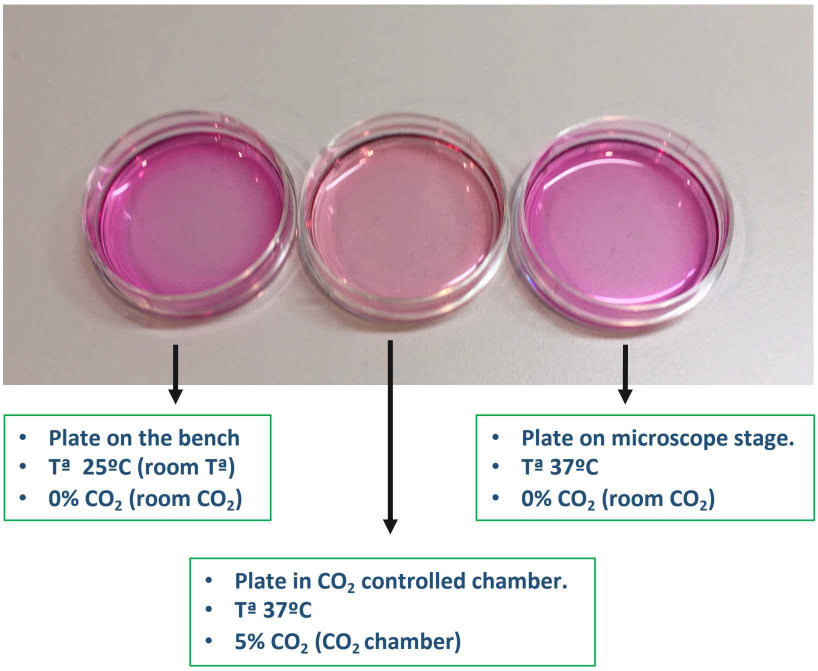

Supplement: Supplementary file 1 [file ijms-22-10876-s001.zip › ijms-1395538-supplementary.pdf]
